# Characteristics of Trailer Thermal Environment during Commercial Swine Transport Managed under U.S. Industry Guidelines

**DOI:** 10.3390/ani5020226

**Published:** 2015-04-10

**Authors:** Yijie Xiong, Angela Green, Richard S. Gates

**Affiliations:** Agricultural and Biological Engineering, University of Illinois at Urbana-Champaign, 1304 W. Pennsylvania Ave, Urbana, IL 61801, USA; E-Mails: angelag@illinois.edu (A.G.); rsgates@illinois.edu (R.G.)

**Keywords:** pigs, management, husbandry, heat stress, cold stress, welfare

## Abstract

**Simple Summary:**

Temperature and thermal conditions of the interior of a swine trailer during transport were monitored over a broad range of outdoor conditions (34 trips total) managed according to industry best practice (Transport Quality Assurance (TQA) guidelines (NPB, 2008)). For the outdoor temperature range of 5 °C (40 °F) to 27 °C (80 °F), generally acceptable trailer thermal conditions were observed according to the TQA. Beyond this outdoor temperature range, undesirable conditions within the trailer were prevalent. Areas for potential improvement in transport management were identified. Stops resulted in rapid increases in temperature, which could be beneficial during cooler outdoor temperatures, but detrimental for warmer outdoor temperatures.

**Abstract:**

Transport is a critical factor in modern pork production and can seriously affect swine welfare. While previous research has explored thermal conditions during transport, the impact of extreme weather conditions on the trailer thermal environment under industry practices has not been well documented; and the critical factors impacting microclimate are not well understood. To assess the trailer microclimate during transport events, an instrumentation system was designed and installed at the central ceiling level, pig level and floor-level in each of six zones inside a commercial swine trailer. Transport environmental data from 34 monitoring trips (approximately 1–4 h in duration each) were collected from May, 2012, to February, 2013, with trailer management corresponding to the National Pork Board Transport Quality Assurance (TQA) guidelines in 31 of these trips. According to the TQA guidelines, for outdoor temperature ranging from 5 °C (40 °F) to 27 °C (80 °F), acceptable thermal conditions were observed based on the criteria that no more than 10% of the trip duration was above 35 °C (95 °F) or below 0 °C (32 °F). Recommended bedding, boarding and water application were sufficient in this range. Measurements support relaxing boarding guidelines for moderate outdoor conditions, as this did not result in less desirable conditions. Pigs experienced extended undesirable thermal conditions for outdoor temperatures above 27 °C (80 °F) or below 5 °C (40 °F), meriting a recommendation for further assessment of bedding, boarding and water application guidelines for extreme outdoor temperatures. An Emergency Livestock Weather Safety Index (LWSI) condition was observed inside the trailer when outdoor temperature exceeded 10 °C (50 °F); although the validity of LWSI to indicate heat stress for pigs during transport is not well established. Extreme pig surface temperatures in the rear and middle zones of the trailer were more frequently experienced than in the front zones, and the few observations of pigs dead or down upon arrival were noted in these zones. Observations indicate that arranging boarding placement may alter the ventilation patterns inside the trailer.

## 1. Introduction

Transport is an essential and critical factor in modern pork production. During transport, pigs experience many undesirable factors and potential weather extremes. Factors that impact the pig’s well-being during transportation include: space allowance and loading density [1,2], transport duration [3], trailer design [4,5,6], handling methods [7] and trailer management [1,5,8,9]. Specifically, great challenges for maintaining thermally-acceptable conditions for pigs have been reported [4,10,11,12], with trailer conditions that include heat stress, cold stress and reduced air quality. Failure to maintain an appropriate thermal environment range during transport can result in increased mortality, decreased product quality, reduced overall production efficiency and compromised animal welfare [2,8,13,14,15,16].

About 200 million pigs are transported annually in commercial over-the-road vehicles in the USA, with approximately 500,000 pigs (0.25%) reported as down or dead on arrival (DOD) [17,18,19]. One study reported that 70% of deaths occurred during transport and that 30% died shortly after arrival [20]. This is a serious economic loss and welfare concern to the swine industry. Previous studies have indicated that the rates of DOD animals increase as the outdoor conditions approach extreme cold or hot [2,8,17,18,19].

The thermal environment of a trailer includes temperature, relative humidity and air circulation and velocity, all of which vary within different sections of a trailer. The trailer thermal environment is not generally actively controlled and is affected by a number of factors, including outdoor temperature, ventilation rate, occupant contribution to thermal load and spatial density, trailer design and transport speed and duration [4,8,16,21]. The document [22] reported that external environment is more critical than space allowance on in-transit loss. [9] found that when the trailer was stationary during loading or waiting at the abattoir, the greatest temperature extremes inside the trailer were observed, which created a greater incidence of thermal stress experienced by the pigs.

Based on previous studies conducted with animal transport trailers, fresh air is expected to enter in the rear and exit towards the middle to the front of the trailer [9,21,23,24]. This ventilation pattern is expected from the understanding of the pressure distributions on the outside of a moving trailer, with lower pressure inside the back and higher pressure towards the front, which drives air into the trailer.

Reports from studies of conditions in swine transport trailers lack documentation of quantitative thermal characteristics. This may in part be omitted because of the challenging nature of acquiring measurements and partially from a lack of understanding of the relation between (uncontrollable) external factors, interior thermal conditions and the few management tools that are available. To address this challenge, a set of Transport Quality Assurance (TQA) management guidelines developed by the National Pork Board is currently applied by the pork industry [25].

The TQA guidelines include recommendations (Table 1) for trailer boarding (the amount of covering of trailer openings) and bedding (presence and depth of a substrate, such as wood shavings) that vary with outdoor temperature. Changes in boarding, in principle, should result in changes in net ventilation of the trailer during transport, although variations in boarding patterns (open toward the front, or the rear, or uniformly along the trailer sides) are not addressed in the TQA. Bedding provides potential insulative effects for the pigs during cold weather and increases footing for the pigs while moving into and out of the trailer, although the likelihood of frozen bedding during extreme cold weather exists.

**Table 1:** Transport Quality Assurance (TQA) guidelines for truck setup procedures during temperature extremes for market pigs [25].

Outdoor Temperature (°C)	Bedding	Boarding (side-slats)	
<−12	Heavy	90% Closed	10% Open *
−12~−6	Medium	75% Closed	25% Open *
−7~3	Medium	50% Closed	50% Open
4~9	Light	25% Closed	75% Open
>10	Light **	0% Closed	100% Open

* Minimum openings are needed for ventilation, even in the coldest weather; ** consider using wet bedding if it is not too humid and trucks are moving.

The evaluation and management of temperature and humidity in the trailer has previously been explored for variations in the macro-environment, such as collecting single measurements from near the ceiling within the various compartments of the trailer [19]. These central measures did not account for spatial variability within the trailer, and micro-environment and uneven air distribution near the pig level may have a significant impact on individual animals. To better characterize the range in thermal environments experienced by trailered pigs during hot, mild and cold weather, the National Pork Board commissioned an observational study [26]. This paper addresses two objectives related to that study:

(1) To develop an instrumentation system to quantify the thermal environment encountered in a loaded swine transport trailer during hot, mild and cold weather.
(2) To characterize the thermal environment encountered by pigs in a commercial swine trailer managed based on industry best practice guidelines (TQA) during hot, mild and cold transport conditions typical of the Midwestern USA.

## 2. Materials and Methods

### 2.1. Trailer Environment Monitoring System

#### 2.1.1. Trailer Description and System Overview

**Figure 1:**
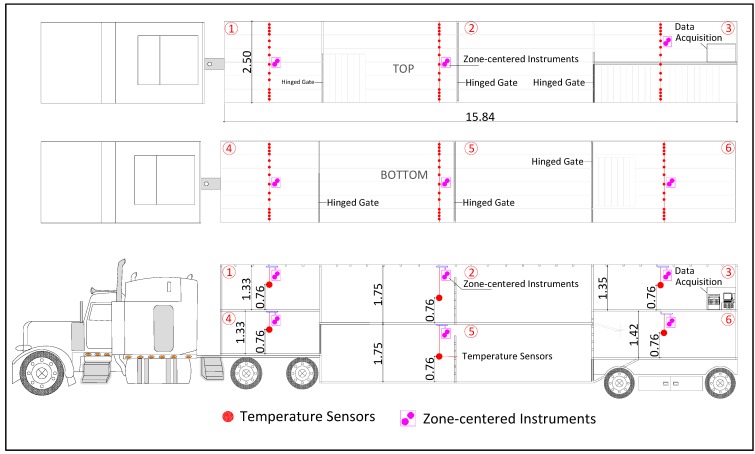
Trailer schematic. The top and bottom plan view of the trailer illustrates the horizontal distribution of sensors within each zone, and the left-side elevation view of the six zones illustrates the vertical distribution of sensors used for pig-level air temperature, surface temperature and zone-centered temperature/relative humidity.

A newly-fabricated commercial swine trailer (Model No. PSDCL-420P with customer-selected features, Wilson Trailer Company, Sioux City, IA, USA) and a loading capacity of 34,020 kg (equivalent to 170–175 market-weight pigs) was used (Figure 1). The trailer overall dimensions were 15.84 m long *×* 2.50 m wide *×* 3.50 m high (from the top to the drop-center). Large (15 *×* 25 cm) and small (7 *×* 14 cm) side-openings were punched identically on the trailer’s outside surface to provide air circulation. The internal trailer space was divided into two levels with three zones on each level (numbered from 1 to 3 on the top level and 4 to 6 on the bottom level, from the front to the back, to identify positions in the trailer).

Each of the six zones was equipped with a set of sensors to measure the trailer thermal environment during transport. Zone measurements consisted of the air temperature of a zone cross-section made near the pig level, the zone-centered air temperature and relative humidity, the pigs’ surface temperature and temperature within the floor/bedding environment. These were taken in all six zones of the trailer (upper and lower levels and the front, center and rear). The sensors used in the system are summarized in Table 2. The placement of each sensor within the trailer is demonstrated in Figure 1.

**Table 2:** Instrumentation summary. Environmental conditions were measured with a set of instruments to represent the thermal environment in three dimensions within the trailer by collecting zone-centered conditions, pig-level air temperature, pig exposed surface temperature and floor/bedding conditions.

Measurement	Location	Instrument Type	Model, Manufacturer	Sampling Frequency
Pig-level Air Temperature	14 sensors for each zone; 84 total	Thermistor	10M5351, Honeywell	1 min
Central Air Temperature and Relative Humidity	Centrally placed at ceiling within each zone	Humidity and Temperature Sensor	HMP60, Vaisala	1 min
Pig Surface Temperature	Centrally placed at ceiling within each zone	Infrared Radiometer	Apogee SI-111, Campbell	1 min
Floor/Bedding Environment	On floor/bedding level, scattered throughout trailer	iButton	DS1921G-F5, Maxim	10 min
Data logging	Upper rear of trailer	Data logger	CR23X, Campbell	1 min

#### 2.1.2. Instrumentation for Environment Monitoring

The instrumentation system was designed for straightforward installation and removal during each monitoring trip, reliable data collection, simple adjustment and minimal labor for periodic monitoring over the course of a year [27]. The data acquisition center, located at the top rear zone of the trailer, consisted of a desktop computer, data logger (Table 2) and three compatible relay multiplexers (Model AM16/32, Campbell Scientific, Inc., Logan, UT, USA). Custom connectors and wiring were implemented to connect sensors from each trailer zone with the data acquisition center. Sensor readings were recorded at a sampling frequency of 1 to 10 min during data collection.

Pig-level air temperature was measured using 84 thermistors (Table 2), consisting of a set of 14 sensors per trailer zone, with one set in each of the 6 zones. The thermistors were protected from damage by routing within PVC pipes and reinforced flex conduit hose. Thermistors were placed 0.76 m (30 in) above the trailer floor in all zones (Figure 1), which was approximately 30 cm (12 in) above the pigs to avoid potential sensor damage by the pigs. For each trailer zone, 4 of the sensors were placed at a 10.2-cm (4 in) interval near each sidewall, and 6 sensors were evenly distributed at a 24.1-cm (9.5 in) interval toward the middle (Figure 1). Air temperature profiles across key locations in the trailer were measured to assess the thermal environment; air velocity was not measured in this study.

The zone-centered environment was measured by a combination temperature and relative humidity sensor (Table 2), which was installed centrally at the ceiling within each zone. The integrated surface temperature within each trailer zone (representing pig skin surface) was measured by one infrared radiometer (Table 2), located at the central ceiling and facing directly downward.

The floor/bedding temperature was captured by multiple stainless steel encapsulated thermistors with built-in loggers (Table 2) placed on the floor within each of the 6 trailer zones, encased in a protective rubber ball holder (Stuff-A-Ball Dog Toy, Kong Company, Golden, CO, USA) with side slits to allow air circulation. Three to six sensors were randomly placed onto the bedding in each zone at the start of transport to best represent the micro-environment at the floor surface, thus similar to the expected bedding temperature, which would be influenced by both the air and floor surface.

### 2.2. Trailer Pre-Monitoring Setup Procedure

The TQA guidelines recommend boarding of trailer openings and variation in the amount of bedding inside the trailer during cold weather and water application and fans for cooling inside the trailer when available during hot weather [25,27]. In this observational study, trailer boarding, bedding and water application were applied according to the TQA guidelines for 31 trips (Table 3). Additionally, three trips were completed with boarding beyond the TQA recommendation during moderate outdoor temperature.

**Table 3:** Summary of outdoor temperature and trailer management under the TQA guidelines for 31 completed monitoring trips.

Outdoor Temperature (°C (°F))	Trailer Managements			Number of Completed Trips
	Bedding	Boarding	Water Application	
<−12 (10)	Heavy	90% with Bottom Covered		4 *
−12 to −7 (10–19)	Heavy	75% Evenly Distributed		1
−7 to 4 (20–39)	Heavy	50% Evenly Distributed		3
4–9 (40–49)	Heavy	25% Evenly Distributed		3
10–20 (50–69)	Medium	0%		6
21–26 (70–79)	Medium	0%		2
27–31 (80–89)	Medium	0%	Water application after loading	3
	Medium	0%	Water application during loading	3
	Light	0%	None	1
>32 (90)	Light	0%	Water application after loading	3
	Light	0%	Water application during loading	2
Total				31

* For the outdoor temperature range <−12 °C (10 °F), one trip experienced thermistor failures and was excluded from analysis involving pig-level air temperatures.

The same driver operated the truck and trailer and managed the animals and trailer configurations according to TQA guidelines, or a modification of the guidelines, during all monitoring trips. Each trip was conducted with full loading capacity (170–175 market-weight pigs at 127–136 kg each); with the total trailer floor space of 79.2 m^2^, the loading density was 275–300 kg/m^2^.

### 2.3. Monitoring, Documentation and Data Analysis

**Figure 2:**
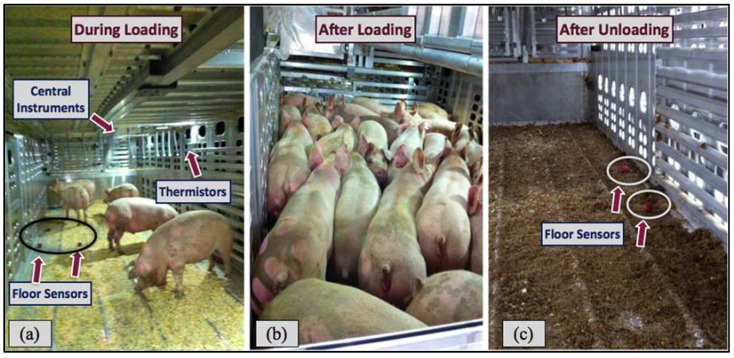
Trailer interior conditions with sensors deployed and pigs on the trailer:(**a**) during loading; (**b**) after loading, before transport; and (**c**) after unloading.

In this study, each monitoring trip was summarized by time periods from before loading the first pig onto the trailer until after the last pig was unloaded (Figure 2). Time periods were defined as: before loading, before transport (loading, water application, waiting at the barn), during transport (between the barn and the destination), after transport (waiting prior to unloading, with and without water application and/or with fans, unloading) and after unloading. During each trip, local weather was monitored and documented for each time period, and any pigs dead or down (DOD) upon arrival were documented with their location of the corresponding trailer zone. External environmental data, including outdoor temperature, relative humidity and wet bulb temperature, were obtained from a local national weather station database based on GPS coordinates during the trip.

The outdoor temperature that occurred in the middle of the transport period was used to categorize the correlative outdoor temperature range of each monitoring trip. Outdoor temperature documented at the start of the loading period was used to determine water application and boarding.

### 2.4. Data Quality Assurance

The accuracy of all sensors deployed was verified in a laboratory environment prior to deployment, over a temperature range from −15 °C (5 °F) to 45 °C (113 °F), using an environmentally-controlled chamber and a NIST (National Institute of Science and Technology)-certified reference temperature device (NT213, Rotronic AG, Bassersdorf, Switzerland). Any sensor not conforming to the manufacturer specifications was calibrated to correct for errors in measurement.

A series of filtering methods was applied to the trailer environmental dataset to remove erroneous data without compromising environmental measures. Air temperature data were filtered to remove gross sensor failures and data outliers [27].

### 2.5. Analysis of Overall Characteristics of Trailer Thermal Environmental Data

#### 2.5.1. Overall Thermal Conditions

To measure the air temperature that describes the thermal micro-environment experienced by the animal, sensors are generally recommended to be located as close to the animal level as practical (e.g., [26,28]). Thus, the pig-level air temperatures and zone-centered environmental dataset were processed and categorized to classify the thermal environment during transport, to calculate the duration of exposure and to document the occurrences of extreme interior trailer temperatures. Thermal comfort conditions were categorized into seven specific trailer internal temperature categories: extreme cold (T_in_ < −15 °C (5 °F)), cold (−15 °C < T_in_ < 0 °C (5 °F < T_in_ < 32 °F)), cool (0 °C < T_in_ < 18 °C (32 °F < T_in_ < 64 °F)), thermoneutral (18 °C < T_in_ < 25 °C (65 °F < T_in_ < 77 °F)), warm (25 °C < T_in_ < 30 °C (77 °F < T_in_ < 86 °F)), hot (30 °C < T_in_ < 35 °C (86 °F < T_in_ < 95 °F)), and extreme hot (T_in_ > 35 °C (95 °F)).

The Temperature-Humidity Index (THI) was processed with Equation (1) [29], using the zone-centered air temperature (converted to °F for THI calculation) and relative humidity (RH) measured in each of the six zones.

(1) $$THI=0.8T_{db}+RH\left(T_{db}-14.4\right)+46.4$$

THI was used to classify the occurrences of the Livestock Weather and Safety Index (LWSI, (LCI 1970)), which are categorized into four categories: normal (THI ≤ 74), alert (75 ≤ THI ≤ 78), danger (79 ≤ THI ≤ 83) and emergency (THI ≥ 84) [29,30,31]. If an interior temperature or LWSI condition occurred at any time and any location during the transport with pigs on the trailer, then this occurrence was counted and tabulated for each of the temperature or LWSI categories. In the frequency table developed, each cell corresponding to a given temperature or LWSI range for each outdoor condition was colored, and the upper number inside each cell indicates the number of trips in which this condition was recorded. A single trip could experience multiple ranges of thermal conditions or LWSI conditions, and all of the conditions encountered were counted for each trip. The frequency of occurrence for each condition was represented by the percentage of the total occurrences for that monitoring trip. The bottom number inside each cell represents the frequency range of trip observations for that condition. For example, a range of 0%–10% would indicate that at least one trip had no observations in that category and at least one had 10% of the observations in that category. For LWSI conditions, the frequency of occurrences was only processed for trips that encountered danger or emergency LWSI conditions.

#### 2.5.2. Zone-Specific Surface Temperature Measurement

Pig surface temperature data were analyzed to represent the occurrences of the extreme thermal conditions experienced by the pigs and to identify problem zones inside the trailer. For each time period, the extreme (maximum and minimum) pig surface temperatures were identified, and the corresponding trailer zones within which these conditions occurred were documented. For each trailer zone, the analysis was performed as follows: the occurrences of trips for which the extreme pig surface temperatures occurred were counted, and the frequency of occurrence of the extreme pig surface temperatures in a specific trailer zone was computed as percentage values over the total number of trips evaluated.

#### 2.5.3. Effects of Boarding Percentage on Pig Surface Temperature

The potential effects of boarding percentages (varying from 0% to 90% coverage) on pig surface temperature were represented by the relationship between pig surface temperature, variable trailer boarding percentages and the corresponding zone-centered air temperature. The minimum, maximum and average pig surface temperature, the corresponding boarding percentage for each monitoring trip and the corresponding zone-centered air temperature in the same zone that the extreme (maximum and minimum) surface temperature occurred were documented for each transport period. A Fisher’s LSD mean separation was conducted to test for any differences between the means of different boarding percentages on temperature measurements. The effects of any boarding percentage were considered significant at α = 0.05.

#### 2.5.4. Effects of Short Breaks during Transport on Trailer Thermal Environment

During each monitoring trip, the trailer operator may stop intermittently for short breaks, the duration of which may range from a few minutes to approximately an hour. To explore the potential effects of such short breaks on the trailer thermal environment, each trailer zone-centered air temperature and the outdoor temperature were compared against the elapsed time of the short breaks.

## 3. Results and Discussion

### 3.1. Monitoring Trip Completion Summary

With trailer management based on the TQA guidelines, 31 monitoring trips were completed from May, 2012, to February, 2013. One trip experienced thermistor failures and was excluded from pig-level air temperature analysis. Table 3 provides the completion summary of these 31 trips corresponding to specific outdoor temperature ranges. Three additional trips were completed with 50% boarding in the 4–9 °C (40–49 °F) range, modified from the TQA guidelines, which specify 25% boarding, evenly distributed.

### 3.2. Overall Characteristics of Trailer Thermal Environment during Commercial Swine Transport

#### 3.2.1. Overall Dead or Down Pigs Summary

During the 31 monitoring trips, a total of four pigs were documented dead or down (DOD) upon arrival at the abattoir (approximately 0.05% of the total pigs transported). The occurrences of DOD were not concentrated for any outdoor condition or management strategy (Table 4). Of the four trips with DOD pigs, one of the dead occurrences was in Zone 6 (bottom rear) and two were in Zone 2 (top middle), while for one trip, it was not possible to identify the corresponding location for the down pig during transport.

**Table 4:** Summary of pigs dead or down (DOD) for all monitoring trips with the trailer managed according to TQA guidelines. All pigs were visually inspected by the trailer operator to confirm the status of no signs of health or behavior abnormalities prior to loading onto the trailer. Pigs with compromised conditions were rejected before they stepped onto the trailer.

Outdoor Temperature (°C (°F))	Trailer Management	Transport Duration (h)	Pig Surface Temperature		DOD * (n)	Location
			min (°C)	max (°C)		
4–9 (40–49)	Boarding, 25% evenly distributed	2.5 and 3.0	14.3 and 19.6	30.7 and 34.5	2^A, 1^	Zone 2
27–31 (80–89)	No water application	1.6	30.5	39.4	1^B^	NA ^2^
>32 (90)	Water application, during loading	3.1	15.5	40.2	1^A^	Zone 6

* For the value in this column, the superscript A indicates that it was a dead pig and the superscript B indicates that it was a down pig; ^1^ these two dead pigs were observed in different trips; ^2^ the pig was able to walk at the beginning of the unloading period and was documented as down when it could no longer stand on its own, thus its original location was unable to be identified.

#### 3.2.2. Overall Thermal Conditions

The assessment of trailer environment was classified by thermal comfort categories based on pig-level air temperature measurements, the duration of exposure and occurrences of interior trailer temperatures (Table 5). A total of 33 trips were evaluated, of which 30 trips followed TQA guidelines (Table 3), and the three others having outdoor temperature ranging from 4–9 °C (40–49 °F) utilized more boarding than TQA guidelines. The Livestock Weather Safety Index (LWSI) was utilized for combining thermal environmental effects, despite its development for predicting heat stress in cattle, since no pig-based options were identified. Although the categories of LWSI may not truly reflect emergency or danger status for pigs during transport, they do provide a comparative assessment of THI within the trailer, while neglecting the effects of air velocity.

**Table 5:** Assessment of trailer environment based on categorizing all 84 pig-level air temperature measurements and their locations into thermal comfort classifications for all transport durations with pigs on the trailer. Data include 33 monitoring trips during hot, mild and cold outdoor conditions. A colored block indicates that the condition occurred at some point during one of the trips monitored. The top number inside the colored block indicates the number of trips experiencing this condition. A single trip may experience multiple ranges of thermal conditions. The bottom number represents the percentage of duration for that condition, with the range covering all trips for the given arrangement.

Temperature (°C)(°F)	Trailer Management			Completed Trips	Thermal Categories						
	Bedding	Boarding	Water Application		Extreme Cold^1^	Cold^2^	Cool^3^	Thermo-neutral^4^	Warm^5^	Hot^6^	Extreme Hot^7^
< -12 (10)	Heavy	90%, with bottom open		3	3	3	3	1			
					(0.1–3.4%)	(15.1–53.7%)	(43.3–83.2%)	(1.6%)			
-12 ~ -7 (10-19)		75%, Evenly Distributed		1	1	1	1	1			
					(0.5%)	(43.2%)	(55.6%)	(0.4%)			
-7 ~ 4 (20-39)	Medium	50%, Evenly Distributed		3		2	3	2			
						(0.4–10.2%)	(89.8–91.4%)	(8.2–9.0%)			
4 - 9 (40-49)	Medium	25%, Evenly Distributed		3			3	2			
							(87.4–100%)	(3.5–12.6%)			
		50%, Evenly Distributed		3		1	3	3			
						(0.3%)	(84.4–98.5%)	(1.2–15.6%)			
10 - 20 (50-69)	Medium		None	6			6	6	5	1	
							(0.2–97.1%)	(2.9–91.4%)	(0.1–8.4%)	(0.2%)	
21 - 27 (70-79)	Medium		None	2			1	2	2	1	
							(0.7%)	(69–93.4%)	(5.9–30.4%)	(0.6%)	
27 - 32 (80-89)	Medium		After Loading	3			1	3	3	2	1
							(1.0%)	(7.3–82.9%)	(16.1–70.3%)	(3.9–41.1%)	(24.1%)
			During Loading	3			1	3	3	3	
							(1.8%)	(1.0–24.1%)	(50.4–98.6%)	(0.4–25.5%)	
			None	1			1	1	1	1	
							(0.4%)	(1.8%)	(67.0%)	(30.8%)	
>32 (90)	Light		After Loading	3				2	3	3	3
								(0.1–0.3%)	(0.7–1.7%)	(57.9–79.4%)	(19.3–41.0%)
			During Loading	2				2	2	2	2
								(0.4–0.5%)	(4.3–4.7%)	(26.3–32.6%)	(62.7–68.5%)

^1^ Extreme cold: T_in_ < −15 °C (5 °F); ^2^ cold: −15 °C (5 °F) < T_in_ < 0 °C (32 °F); ^3^ cool: 0 °C (32 °F) < T < 18 °C (62 °F); ^4^ thermoneutral: 18 °C (62 °F) < T < 25 °C (77 °F); ^5^ warm: 25 °C (77 °F) < T < 30 °C (86 °F); ^6^ hot: 30 °C (86 °F) < T < 35 °C (95 °F); ^7^ extreme hot: T > 35 °C (95 °F).

For outdoor temperatures greater than 10 °C (50 °F), there were 20 trips evaluated (Table 5), of which pigs experienced extreme hot conditions in six trips. Specifically, for outdoor temperatures above 32 °C (90 °F), a high frequency of hot conditions (ranging from 62.7% to 68.5%) was observed, which indicates that more than 60% of the time × location measurements recorded extreme hot conditions (above 35 °C (95 °F)) on the trailer during these trips. For outdoor temperatures below 10 °C (50 °F), there were 13 trips evaluated (Table 5), of which pigs experienced extreme cold conditions for four trips, ranging from 0.1% to 3.4% of all time × location measurements.

When outdoor temperature exceeded 21 °C (70 °F), Emergency LWSI (Table 6) conditions were reported for nine of the 14 trips, with no extreme hot temperature reported inside the trailer for four of those emergency trips.

**Table 6:** Assessment of trailer environment based on categorizing all pig-level air temperature and zone-centered environmental measurements and their locations into LWSI categories for all transport durations when pigs were on the trailer. Data included 20 monitoring trips during temperatures above 10 °C. A colored block indicates that the condition occurred at some point during one of the trips monitored. The top number inside the colored block indicates the number of trips experiencing this condition. A single trip may experience multiple ranges of thermal comfort. The bottom number of the last two columns represents the percentage of the duration for that condition, with the range covering all trips for the given arrangement. THI, Temperature-Humidity Index.

Temperature (°C)(°F)	Water Application*	Bedding Arrangement	Completed Trips	Livestock Weather Safety Index (LWSI) Categories			
				Normal^1^	Alert^2^	Danger^3^	Emergency^4^
10-20 (50-69)	None	Medium	6	6	2	1	1
						(0.3%)	(0.1%)
21-26 (70-79)	None	Medium	2	2	2	1	
						(1.2%)	
26-32 (80-89)	After Loading	Medium	3	3	3	3	2
						(1.3 - 32.8%)	(0.8 - 3.9%)
	During Loading	Medium	3	3	3	3	2
						(1.0 - 35.6%)	(0.2 - 0.5%)
	None	Medium	1	1	1	1	
						(1.3%)	
>32 (90)	After Loading	Light	3	3	3	3	3
						(47.7 - 74.8%)	(0.6 - 23.8%)
	During Loading	Light	2	2	2	2	2
						(32.6 - 61.9%)	(33.0 - 63.7%)

* No boarding was applied during warm temperatures; ^1^ normal: THI < 74; ^2^ alert: 74 < THI < 78; ^3^ danger: 78 < THI < 84; ^4^ emergency: THI > 84.

Based on the results of this overall summary, pigs experienced undesirable temperature conditions when outdoor temperature exceeded 27 °C (80 °F) or was below 5 °C (40 °F). Compared to cold conditions during transport, pigs had more potential to experience heat stress for greater durations when outdoor temperatures became extreme during hot weather conditions.

#### 3.2.3. Zone-Specific Pig Surface Temperature inside the Trailer

**Figure 3:**
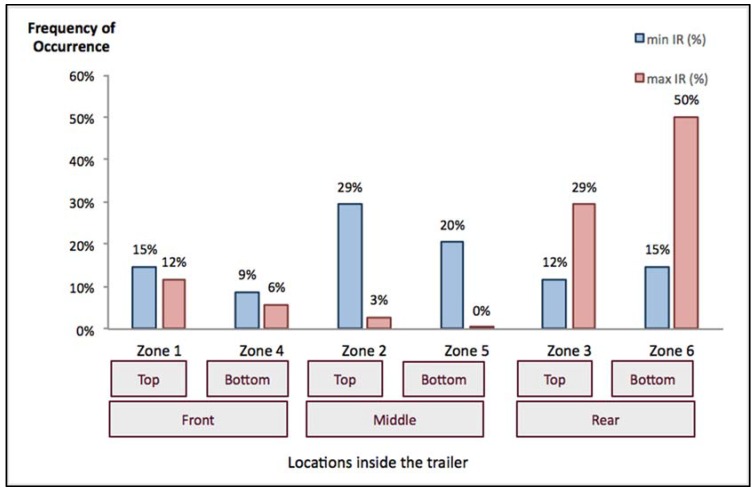
Location and frequency of occurrence of extreme pig surface temperatures within the trailer for 34 monitoring trips over outdoor temperatures ranging from −14 to 38 °C (7 to 100 °F).

The dry bulb temperature difference between the pig’s skin and the air is the major contributor to the convective heat loss from the pigs to the environment. The greater the temperature differences, the greater is the convective component’s contribution to the overall heat loss for a pig, regardless of outdoor temperatures. The maximum and minimum pig surface temperatures captured by the infrared radiometer (IR) sensors represent the potential heat loss range encountered by the pigs and indicate the locations within the trailer where the most extreme thermal environment was experienced, which might compromise the performance of the pigs. Figure 3 demonstrates the frequency of occurrence for the zone location of the extreme pig surface temperatures for 34 monitoring trips.

Maximum surface temperature indicates the zone within the trailer where pigs must lose the most heat to the environment for thermoregulation during hot weather and where pigs may potentially be experiencing a warm or thermoneutral environment in cold weather. Over all of the observations, a total of 79% of the trips with a maximum surface temperature were observed in trailer rear sections (Zones 3 and 6). For other locations inside the trailer, the frequencies of occurrence for the maximum surface temperature were observed under 20% within each zone.

By contrast, the minimum surface temperature potentially represents the zone within the trailer where pigs needed to conserve heat, but were instead losing it to the environment at a rate that drops their surface temperature during cold weather and where pigs were most successfully handling heat stress during the hot weather. For locations of the minimum pig surface temperature, a total of 49% of the trips with the minimum pig surface temperatures was observed in the middle sections of the trailer (Zones 2 and 5). For other trailer zones, the minimum pig surface temperatures were reported in the trailer front sections for 24% and in the rear sections for 27% of the trips evaluated.

This analysis of the most extreme conditions within the trailer revealed that the pigs in the rear sections of the trailer experienced a higher frequency of the occurrence of the maximum surface temperatures, and those in the middle sections encountered a higher frequency of occurrence of the minimum surface temperatures, regardless of the outdoor air temperatures. The same general trend was observed when this analysis was broken out by specific outdoor temperature ranges and boarding arrangements. Thus, the middle sections of this trailer, when fully loaded, appeared to be the most ventilated regardless of weather, transport speed or season. Conversely, the rear sections appeared to be the least ventilated. This differs from findings in other studies, where the rear of the trailer would be the ventilation inlet and the front would be the exhaust [9,21,24].

Previous studies have indicated that compared to cold stress, heat stress has the potential to increase mortality and production efficiency losses, especially under hot conditions [32,33]. As indicated in Table 5, while only a small portion (<30% for 63% of the trips evaluated) of transport durations experienced thermoneutral conditions (18 °C < T_in_ < 25 °C (65 °F < T_in_ < 77 °F)), the majority of the trailer experienced warm through hot conditions (T_in_ > 25 °C (77 °F)). By observing that the majority of the maximum surface temperatures occurred in the rear sections of the trailer, there appears to be a great concern for heat stress during road transport. Realizing that the cooling effects of ventilation or water application were not uniform throughout the trailer, one approach to alleviate this variability may be to focus on the rear sections of the trailer when applying cooling methods to the trailer after transport (*i.e.*, adjusting the location of the trailer to keep the rear sections closer to the fan bank, having more misting nozzles in the rear sections, *etc.*), though this approach was not tested in this study.

Under cold weather conditions, the minimum temperatures in the trailer middle sections likely indicate that openings in the trailer wall were serving as ventilation inlets. Prior to this study, our assumption was that openings at the back of the trailer served as air inlets and that those towards the front served as outlets. That assumption is not supported in this study, since the rear sections of the trailer experienced a more frequent occurrence of the maximum surface temperatures, as would be expected if that section served as an outlet. Thus, it is possible that by rearranging trailer boarding, we could change the location of air inlets and outlets, potentially encouraging more uniform air distribution inside the trailer. Concentrating more trailer boarding in the middle sections may result in shifting some of the ventilation inlets toward the front or rear to create a more uniform temperature distribution inside the trailer and reducing the risk of increasing concentrated cooler temperatures inside the trailer.

#### 3.2.4. Effects of Trailer Bedding Depth on Skin Surface Temperature

The pig surface temperature extremes were regressed against the corresponding zone-centered air temperatures, revealing a linear relation between the two variables, regardless of the different trailer bedding depths used. If the bedding affects surface temperature, a temperature gradient between the three bedding depths would be expected. However, given the linear relation noted between surface temperature and zone-centered air temperature, there is no evidence to support this notion. The three bedding depths used in these trips did not provide any observable additional thermal insulation to the pigs, as measured by differences in pig surface temperature. This result agrees with a recent study [34], which reported no significant effect on the thermal environment for 3–12 bags of bedding for outdoor temperatures ranging from −13 to 45 °C (8 to 113 °F). In that study, the trailer was divided into four compartments, and five laser thermometers were used to collect pig surface temperature on the flank/side of ten randomly selected pigs during unloading. Their results also showed that the surface temperature of pigs exiting the trailer changed with air temperature and had no dependence on trailer bedding depth.

#### 3.2.5. Effects of Boarding Percentage on Pig Surface Temperature Measurements

Pig surface temperature extremes were regressed against the corresponding zone-centered air temperature as the independent variable and were found to be independent of trailer boarding percentage (see [27]). For minimum surface temperatures at colder outdoor temperatures, the relationship was less distinct, which may be an indication that trailer boarding has an impact under these conditions.

**Figure 4:**
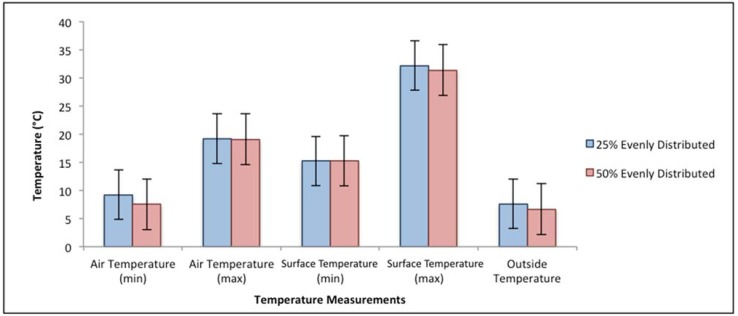
Effects of additional boarding beyond TQA guidelines (50% *vs.* 25% for outdoor temperature range 4–9 °C (40–49 °F), *n* = 3) on maximum and minimum observations for zone-centered air temperature and pig surface temperature. Error bars were included to indicate the standard deviations for each scenario.

The effect of additional boarding beyond TQA guidelines is summarized in Figure 4. Maximum and minimum observations for the response variables are nearly identical for the two boarding configurations, indicating no impact of the additional boarding for this outdoor temperature range.

Results show no statistical difference (*p* = 0.53–0.99 for four mean separations) for max/min zone-centered air temperatures or max/min pig surface temperatures between the two boarding percentages. By looking at the minimum and maximum temperature measurements (either pig-level air temperature or pig surface temperature), additional boarding percentage did not increase the maximum temperatures, nor did it increase the minimum temperatures in the trailer, while less boarding percentage did not lower the minimum temperatures inside the trailer. This is also supported by the frequency of the occurrences of the thermal conditions analysis. For outdoor temperature range of −7 to 9 °C (20 to 49 °F), the majority of the trailer thermal environment stayed within cool conditions (0 °C < T_in_ < 18 °C (32 °F < T_in_ < 65 °F)), and only a small portion of the transport (0.2% to 10.2%) encountered cold conditions (−15 °C < T_in_ < 0 °C (5 °F < T_in_ <32 °F)).

The results suggest that the TQA boarding guidelines may be relaxed to allow some additional flexibility and discretion of the trailer operator without compromising the thermal environment inside the trailer for the outdoor temperature range of −7 to 9 °C (20 to 49 °F). The effect of modifying the amount of boarding for other temperature ranges was not fully explored in this study and is recommended in future work to better understand the implication of boarding practices.

#### 3.2.6. Effects of Short Breaks during Transport on Trailer Thermal Environment

Stopping for breaks resulted in rapid temperature increases inside the trailer. An approximately 1 °C (1.8 °F) temperature rise in a minute and a 3–4 °C (5–7 °F) rise in 5 min were observed. Based on observations during monitoring trips, this situation happened in cases when the trailer stopped for short breaks of more than 5 min. For hot weather conditions, even when the total temperature rise within the trailer was limited to 1 to 2 °C (2 to 4 °F), thermal conditions shifted to a more dangerous level (*i.e.*, from hot conditions to extreme hot conditions). For mild to cold weather conditions, higher temperature rises (3–4 °C (5–7 °F) within 5 min) were observed throughout the trailer, which alleviated the thermal condition from the previous transport section to a milder level (*i.e.*, from cold conditions to cool conditions).

Based on these results, brief stops of the trailer should be limited during hot weather conditions, while stopping during cold conditions may have a benefit for alleviating some of the cold conditions. The implications of this approach were not explored in this study, such as impacts on gas concentrations or animal fatigue.

## 4. Conclusions

A total of 34 trips were monitored with a commercial trucking company hauling finished pigs to market. Thirty-one of these trips were managed according to TQA guidelines for bedding, boarding and water application, from May, 2012, to February, 2013. Three additional trips were completed with additional boarding modified from the TQA guidelines. For outdoor temperatures ranging from 5 °C (40 °F) to 27 °C (80 °F), the TQA guidelines were found to provide acceptable trailer thermal conditions. Recommended bedding, boarding and water application were sufficient in this range, and the results indicated that relaxing the boarding guidelines for moderate conditions did not result in less desirable conditions. Pigs experienced undesirable thermal conditions outside this outdoor temperature range, meriting further assessment of bedding, boarding and water application during extreme conditions. A Livestock Weather Safety Index (LWSI) in the emergency category was observed on the trailer when the outdoor temperature exceeded 10 °C. Trailer rear zones most frequently resulted in the maximum pig surface temperatures, and middle zones most frequently resulted in the minimum pig surface temperatures, corresponding to the few pigs dead or down upon arrival. Varying boarding levels and distributions show the potential for altering the ventilation patterns within the trailer and merit further exploration as a technique to increase thermal uniformity throughout the trailer by manipulating the location of fresh air inlets and outlets. Stopping for breaks resulted in rapid temperature increases (3–4 °C (5–7 °F) within 5 min) inside the trailer and should be reduced or extremely limited during hot weather conditions, but may have benefits to alleviate some of the freezing conditions during cold weather conditions.
